# Bumble bee (*Bombus impatiens*) survival, pollen usage, and reproduction are not affected by oxalate oxidase at realistic concentrations in American chestnut (*Castanea dentata*) pollen

**DOI:** 10.1007/s11248-021-00263-w

**Published:** 2021-06-10

**Authors:** Andrew E. Newhouse, Anastasia E. Allwine, Allison D. Oakes, Dakota F. Matthews, Scott H. McArt, William A. Powell

**Affiliations:** 1 Department of Environmental and Forest Biology, SUNY College of Environmental Science and Forestry, 1 Forestry Drive, Syracuse, NY 13210 USA; 2 Department of Entomology, Cornell University, 2130 Comstock Hall, Ithaca, NY 14853 USA

**Keywords:** Transgenic, Biotechnology, Biosafety, Risk assessment, GMO

## Abstract

Transgenic American chestnut trees expressing a wheat gene for oxalate oxidase (OxO) can tolerate chestnut blight, but as with any new restoration material, they should be carefully evaluated before being released into the environment. Native pollinators such as bumble bees are of particular interest: *Bombus impatiens* use pollen for both a source of nutrition and a hive building material. Bees are regular visitors to American chestnut flowers and likely contribute to their pollination, so depending on transgene expression in chestnut pollen, they could be exposed to this novel source of OxO during potential restoration efforts. To evaluate the potential risk to bees from OxO exposure, queenless microcolonies of bumble bees were supplied with American chestnut pollen containing one of two concentrations of OxO, or a no-OxO control. Bees in microcolonies exposed to a conservatively estimated field-realistic concentration of OxO in pollen performed similarly to no-OxO controls; there were no significant differences in survival, bee size, pollen use, hive construction activity, or reproduction. A ten-fold increase in OxO concentration resulted in noticeable but non-significant decreases in some measures of pollen usage and reproduction compared to the no-OxO control. These effects are similar to what is often seen when naturally produced secondary metabolites are supplied to bees at unrealistically high concentrations. Along with the presence of OxO in many other environmental sources, these data collectively suggest that oxalate oxidase at field-realistic concentrations in American chestnut pollen is unlikely to present substantial risk to bumble bees.

**Supplementary Information:**

The online version contains supplementary material available at 10.1007/s11248-021-00263-w.

## Introduction

American chestnut trees (*Castanea dentata* [Marsh.] Borkh.) were once prominent features of many eastern US deciduous forests, with notable economic and ecological value. Their range was centered around the Appalachian mountains, and extended from Maine to Mississippi and into southern Ontario, though recent models suggest optimal habitat may be shifting northwards with warming climates (Barnes and Delborne 2019). Primary habitat types consisted of mixed mesic forests and ridgetops (Braun 1950; Wang et al. 2013). These majestic trees were decimated in the twentieth century by chestnut blight, caused by the invasive fungus *Cryphonectria parasitica* ([Murr.]Barr) (Anagnostakis 1987). One of the virulence factors employed by the fungus is oxalic acid (Chen et al. 2010), which kills living American chestnut tissue under the bark. Oxalic acid is also a known toxin to many other organisms including humans (Massey et al. 1993) and bees (Rademacher et al. 2017). At appropriate concentrations, oxalic acid can also be an effective miticide in honey bee hives (Gregorc and Planinc 2002).

Restoring chestnuts to their former woodland habitats has been a priority since soon after the blight was identified (van Fleet 1914; Graves 1940). Restoration efforts are ongoing, but the goal of using traditional breeding to establish American chestnuts with effective blight resistance has been more challenging than initially anticipated (Westbrook et al. 2020b). As part of a modern, multifaceted restoration strategy (Steiner et al. 2017; TACF 2020), American chestnuts have been transformed with a gene from wheat encoding oxalate oxidase (EC 1.2.3.4) (Zhang et al. 2013; Powell et al. 2019; Newhouse et al. 2020). Similar enzymes are found in cereal grains, many other monocots, and some dicots (Laker et al. 1980; Satyapal and Pundir 1993; Molla et al. 2013). Oxalate oxidase (OxO) degrades oxalic acid, yielding carbon dioxide and hydrogen peroxide. This degradation increases the tree’s tolerance to chestnut blight, without directly harming or repelling the fungal organism (Newhouse et al. 2020). This enzyme is effective at protecting American chestnut tissues from the effects of blight (Welch et al. 2007; Newhouse et al. 2014), but as with any new product or trait applied to the environment, it is prudent to consider potential effects on other ecosystem interactions in the chestnut’s habitat.

One topic of current concern is the health of insect pollinators, which may be increasingly threatened by invasive species, parasites, land use changes, climate change, and pesticide use (Williams and Osborne 2009; Potts et al. 2010; Goulson et al. 2015). Even naturally-sourced pesticides can present distinct risks to bee activity and survival (Xavier et al. 2015). Therefore, it is important that risks to pollinators are evaluated on potential restoration material, regardless of the methods or products employed. A recent review of transgenic plant effects on honey bees (Ricroch et al. 2018) concluded that the majority of transgenic plants evaluated to date “do not negatively affect the survival of honey bees and have no potential sublethal effect in controlled laboratory conditions or in field/semifield trials.” (They specified one toxin type, protease inhibitors, whose risk depends on concentration in pollen.)

Bees likely contribute to pollination of chestnuts (Manino et al. 1991; de Oliveira et al. 2001; Hasegawa et al. 2015), and multiple bee species have been observed visiting catkins (male flowers) on American chestnuts and other *Castanea* spp. (Giovanetti and Aronne 2011; Tumminello 2016; Zirkle 2017). In addition to these observed interactions, *Castanea* pollen has been shown to be especially nutritious to bumblebees (Tasei and Aupinel 2008a), so restoring chestnuts to their former habitat may benefit pollinators by providing additional foraging resources.

Several environmental interactions have already been observed or tested experimentally with transgenic chestnuts, including mycorrhizal interactions with chestnut roots (Tourtellot 2013; D’Amico et al. 2015), native seed germination through chestnut leaf litter (Newhouse et al. 2018), insect herbivory on chestnut leaves (Brown et al. 2020), chestnut leaf decomposition rates (Gray 2015), aquatic insect survival and growth on chestnut leaves (Newhouse et al. 2020), and tadpole feeding on aquatic leaf litter (Goldspiel et al. 2019). The overwhelming consensus from these studies is that differences between transgenic chestnuts and non-transgenic controls are either insignificant or smaller than changes resulting from traditional hybrid breeding. These previous studies all involved transgenic chestnut trees, or tissue collected directly from transgenic trees. Due to permit limitations with pollen production in confined field trials, transgenic pollen was not available in quantities required for bee feeding studies, which necessitated use of exogenously supplied purified OxO enzyme mixed with non-transgenic chestnut pollen (see Methods). Similar procedures (i.e. supplying purified secondary metabolites or systemic insecticides in pollen) have been used in previous bee studies (Elston et al. 2013; Arnold et al. 2014; Xavier et al. 2015).

The current study explores potential effects of oxalate oxidase in American chestnut pollen on the survival, size, pollen consumption, hive construction, and reproductive effort of native bumblebees (*Bombus impatiens*). This bee was chosen as it is an abundant generalist pollinator on both wild plants and agricultural crops, and it uses pollen as both a source of nutrition and as a hive building material (Cook et al. 2013; Williams et al. 2014). It has also been specifically observed visiting American chestnut catkins (Tumminello 2016), and hives are commercially available. We employed *B. impatiens* microcolonies as bioassays, each containing 5 worker bees, to allow social interactions and observable reproductive effort in replicated experimental units (Babendreier et al. 2008; Manson and Thomson 2009; Gradish et al. 2013). Microcolonies have been used previously to assess the impact of pollen chemistry on bumble bee performance and survival (Arnold et al. 2014).

Here, in order to assess potential risks posed to bees by OxO in chestnut pollen, we address the following questions: (1) What is the effect of field-realistic concentrations of OxO on bumble bee pollen consumption and colony growth? And (2) what is the effect of field-realistic concentrations of OxO on bumble bee survival?

## Methods

### Microcolony setup

**Fig. 1:**
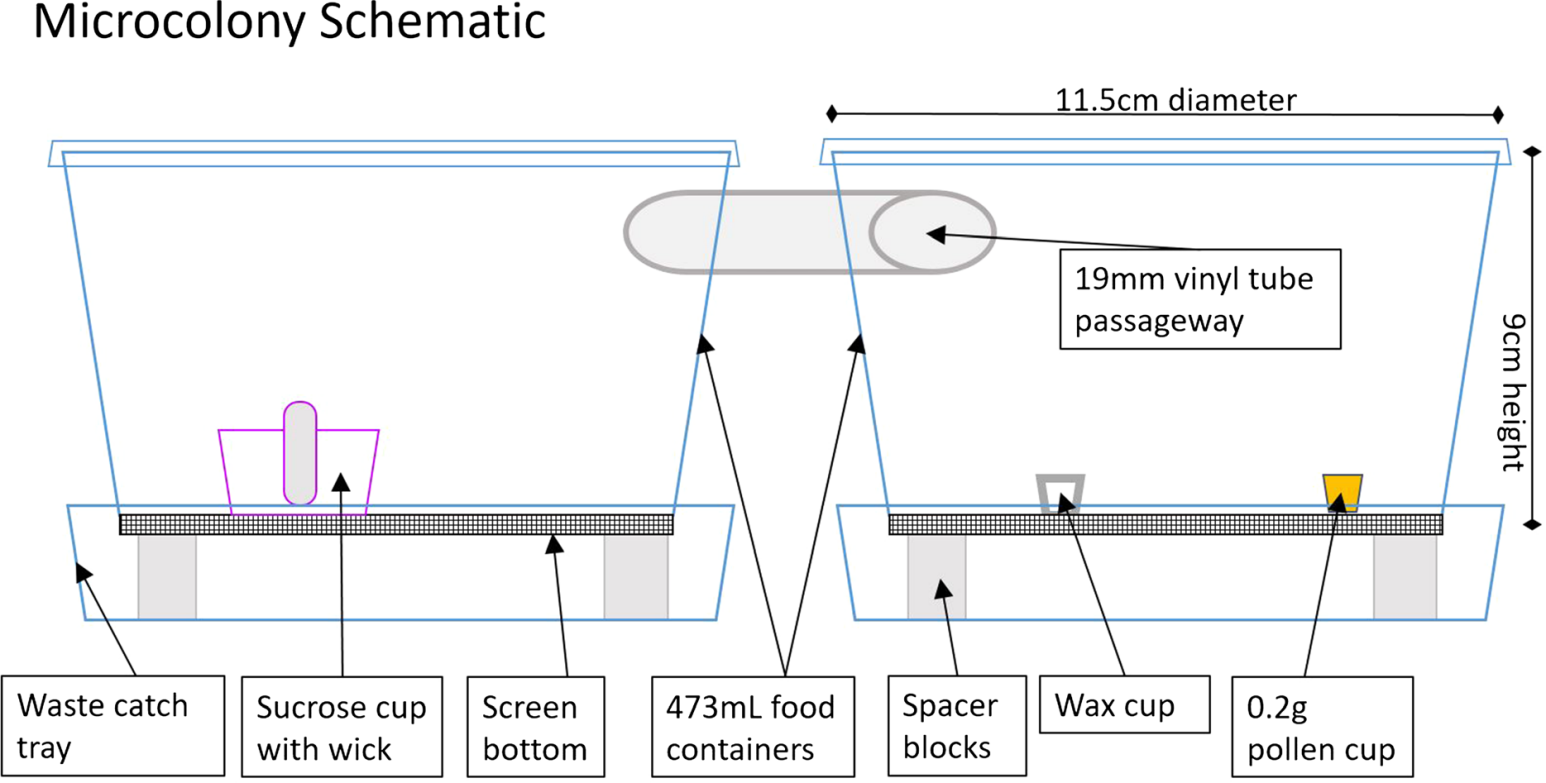
Schematic sketch of microcolony setup

Each microcolony was constructed from two 473 mL plastic take-out food containers, connected by 19 mm diameter vinyl tubing to allow bees to pass between containers (Fig. 1). The bottom of each container was cut out and aluminum window screen was glued in its place, to allow waste to fall through into a catch tray (comprised of a 236 mL plastic food cup, containing spacer blocks to support the larger containers). One of the two 473 mL containers was supplied with a 12 mL capped container of 44% w/v sucrose (accessible to the bees through a cotton wick), and the other was supplied with 0.2 g of pollen in a plastic cup (approx. 5 mm × 5 mm) and a similar-sized cup made of wax to stimulate oviposition (Gradish et al. 2013) and encourage nest building activity. Sucrose solution (44% w/v) was prepared in distilled water, autoclaved, and stored at 4 °C when not in use.

### Pollen collection

Pollen was collected from a Top Mount Pollen Trap (Betterbee, Greenwich, NY), which was installed in a honey bee hive placed near several flowering non-transgenic American chestnut trees in Syracuse, NY. Pollen pellets from this trap were collected daily during and after chestnut flowering season (~ July 2017) and stored at −20 °C after each day’s collection. Before use, pellets were crushed with a mortar and pestle and mixed with sucrose water until a putty-like ball formed, which was stored at −20 °C when not in use. Pollen used for the experiment consisted of approximately 30% chestnut pollen, with the remaining 70% consisting of other mixed flower types. Chestnut pollen pellets were identified before mixing by an orange-gold pellet color unique to this mix, and the ratio was confirmed after mixing by examination with a scanning electron microscope. Nine electron micrographs of the mixed pollen were produced from the same pollen mixture used in the study, and all identifiable pollen grains were coded independently by two observers as either chestnut or non-chestnut.

Pollen supplied for non-experimental purposes (e.g. to source colonies and partially filled microcolonies before observations started) was collected from the same trap after chestnut flowering season (late July), so it consisted of mixed non-chestnut flower pollen from the same geographical location.

### Preparation of pollen treatments with OxO

Pollen treatments were created by adding purified OxO enzyme from barley (Roche Diagnostics, Mannheim, Germany) at two concentrations to sucrose water before mixing with chestnut pollen. For the standard concentration treatment, purified OxO was added to a final concentration of 0.15 µg OxO/mg fresh mixed pollen (equivalent to 1 µg OxO/mg fresh weight of transgenic chestnut pollen; see Discussion). An artificially high concentration treatment was also used, with ten times the standard treatment (1.5 µg OxO/mg pollen). Finally, a no-OxO control was created using the same chestnut pollen mix with no added enzyme. Pollen treatments were assigned arbitrary numbers, so the composition of each treatment was not apparent to observers during the experiment, in order to prevent any potential bias during observations. OxO enzymatic activity was tested before and after the study with a histochemical assay (Dumas et al. 1995) in all treatments. Briefly, this assay involves soaking tissue samples in a solution containing 4-chloro-1-naphthol, which forms a blue-black precipitate in the presence of hydrogen peroxide. If the tissue contains active OxO, oxalic acid in the solution is broken down, yielding hydrogen peroxide and resulting in a distinct color change.

### Source colonies

Three *Bombus impatiens* medium hives (hereafter referred to as “source colonies” and denoted A, B, and C) were purchased from Biobest USA (Leamington, ON, Canada) in August 2017. Newly emerged (< 24 h old) workers were removed from these source colonies daily and sorted into separate microcolonies. Each microcolony received bees from only one source colony, so microcolonies could be blocked by source colony during analysis. If a microcolony was partially filled, it was temporarily supplied with sucrose and non-chestnut pollen. Treated chestnut pollen was supplied and daily observations started as soon as a given microcolony was filled with 5 bees (Gradish et al. 2013). This first day of treatment in a full microcolony was considered Day 1 for all analyses (see Supplemental Table 1 for start dates). Bees from a given source colony were assigned sequentially to each pollen treatment to maintain similar ages in a single microcolony and across all treatments. Due to availability of newly emerged bees, replicate numbers varied slightly among treatments: n = 14, 7, and 6 microcolonies for sources A, B, and C respectively; n = 8, 10, and 9 for no, standard, and high OxO treatments respectively (Table 1).

**Table 1:** Reproductive output. Source colony was a significant factor (*p* ≤ 0.034) for numbers of eggs, larvae, and combined offspring. Row headings (left) show number of replicates for each treatment and source

		Eggs		Larvae		Emerged adults	Combined offspring	
		$\stackrel{-}{x}$± SEM	F, *p*	$\stackrel{-}{x}$± SEM	F, *p*	$\stackrel{-}{x}$± SEM	$\stackrel{-}{x}$± SEM	F, *p*
OxO conc	None(n = 8)	4.75 ± 1.76	1.431, 0.265	2.13 ± 1.72	0.681, 0.518	0.13 ± 0.13	7.00 ± 3.13	1.650, 0.220
	Std(n = 10)	4.60 ± 2.02		2.40 ± 1.06		0.30 ± 0.15	7.30 ± 2.95	
	High(n = 9)	1.78 ± 0.83		0.78 ± 0.47		0 ± 0	2.56 ± 1.25	
Source	A(n = 14)	6.50 ± 1.47	6.842, 0.006*	3.43 ± 1.09	4.086, 0.034*	0.29 ± 0.13	10.2 ± 2.27	8.298, 0.003*
	B(n = 7)	0.57 ± 0.37		0 ± 0		0 ± 0	0.57 ± 0.37	
	C(n = 6)	0.83 ± 0.65		0 ± 0		0 ± 0	0.83 ± 0.65	

Presented as mean ( $\overline{x }$) number of individuals per microcolony ± 1 standard error of the mean (SEM), with F and *p*-values from ANOVA where relevant: * indicates a significant difference for a given offspring type at α = 0.05. OxO concentration was not a significant factor for any reproductive measurement (*p* ≥ 0.220).

### Observations, maintenance, and analysis

Daily observations consisted of counting live bees, assessing pollen consumption (mg/day) and replenishing if necessary, assessing remaining sucrose solution and replenishing if necessary, counting total constructed nectar cells, counting total constructed egg cells, and removing any dead bees. Sucrose (nectar) solution was replenished by adding 10 mL to the cup whenever the remaining quantity fell below 2 mL, and these refills were tracked to observe total nectar consumption in each microcolony. Pollen was replenished (filled up to 0.2 g) whenever remaining quantity fell below 0.07 g. A separate pollen cup of untreated pollen was kept near the microcolonies, emptied and refilled every 6 days, and massed daily to quantify mass lost due to evaporation.

Daily pollen usage was calculated per individual bee, rather than per whole microcolony, to account for individual bee mortality. Most microcolonies (regardless of OxO treatment or source colony) used negligible quantities of pollen after 5 weeks, and the first offspring emerged at day number 36, so pollen use analyses are presented here for Days 1–35. When possible, observations continued until a given microcolony reached 50 days old, when all bees died, when a new adult offspring emerged, or on 23-Dec-2017, whichever came first (end dates and reasons are listed in Supplemental Table 1). At this conclusion or whenever a dead bee was removed, each bee was massed, radial cell (RC) length was measured on both wings, and intertegular (IT) distance was measured, as these parameters have been reported as indicators of overall bee size in previous studies (Tasei and Aupinel 2008b; Cariveau et al. 2016). RC and IT measurements were performed with a digital caliper (iGaging IP54, China, 0.01 mm resolution). Mass of bees and pollen cups was measured on a digital balance (Fisher Science Education ALF203, USA, 0.001 g resolution). When all bees had died or been removed from a given microcolony, all constructed cells were dissected to obtain final counts and masses of larvae and eggs.

### Statistical analysis

We used linear mixed effect models using the lmer function in the lme4 package (Bates et al. 2015) combined with the lmerTest package (Kuznetsova et al. 2017) in R (version 3.6.0) (R Development Core Team 2008) to build mixed-effect models to test for significant differences between treatment and source colony for individual bee mass, IT distance, and radial cell length, with the microcolony set as the random effect. We also used linear models to test for differences between treatment, time, source colony and their interactions with microcolony as the unit of replication for the variables total pollen consumed, nectar use, cells formed, and offspring. We performed a repeated measures ANOVA on pollen use per day, with OxO treatment, source colony, and time as fixed effects. We generated survival plots using the functions survfit, ggsurvplot, survdiff, and pairwise_survdiff in the survminer package (Kassambara and Kosinski 2017), and Cox hazard ratio using the forest_model function in the forestmodel package (Kennedy 2017).

## Results

### Pollen and nectar consumption

According to the pollen evaporation control, newly replenished pollen consistently lost an average of 10% (range 6–14%) of its mass within 24 h after being supplied, and plateaued at 11% (range 9–15%) mass lost after 3–5 days, so 10% evaporation loss was incorporated into daily pollen usage calculations the day after each replenishment. OxO activity was clearly visible in both OxO treatments according to a histochemical test performed after the study, confirming the enzyme was stable and active throughout the experiment.

**Fig. 2:**
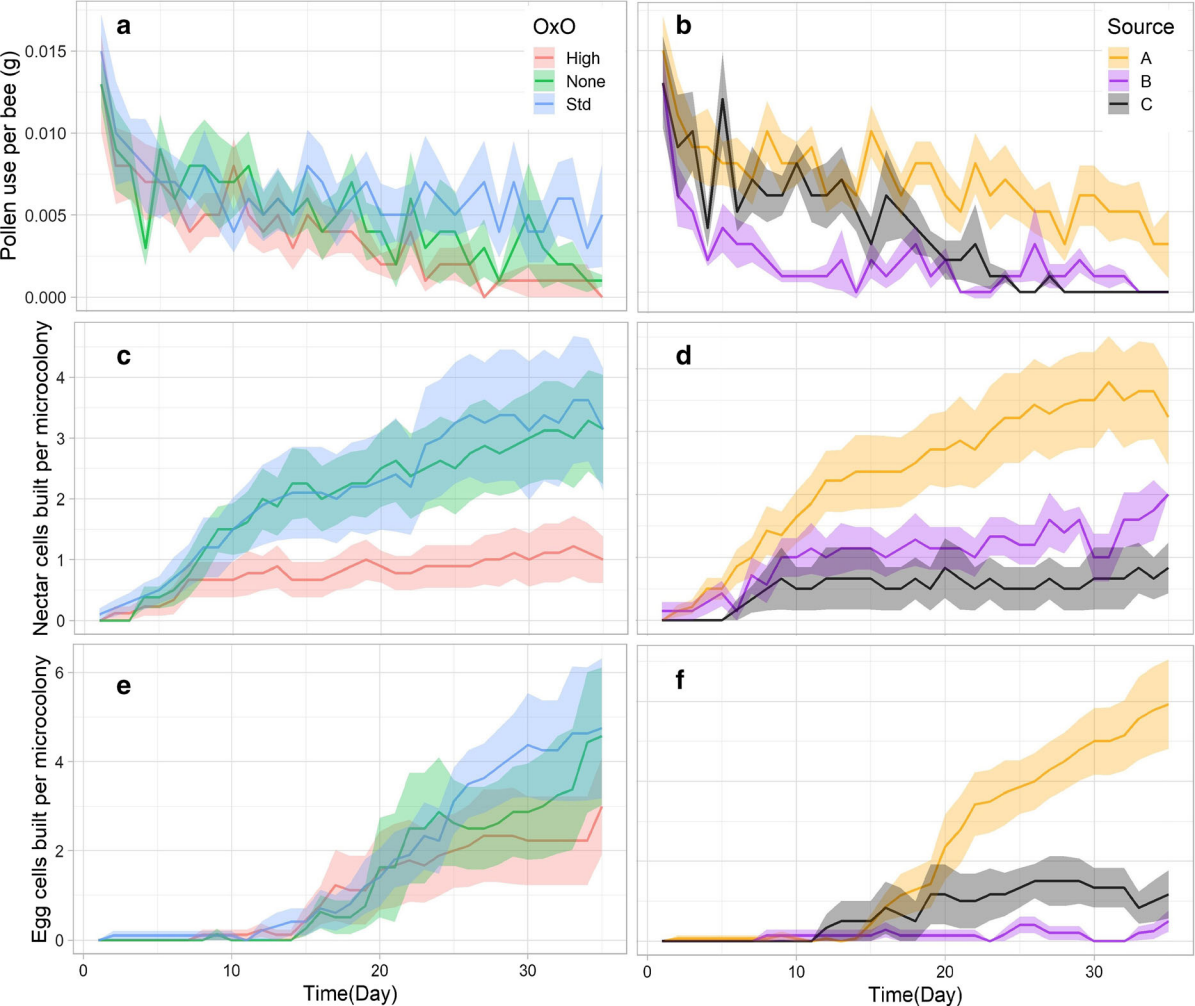
Pollen use per bee over 35 days by OxO treatment (**a**) and source hive (**b**); Nectar cells built per microcolony by OxO treatment (**c**) and source hive (**d**); Egg cells built per microcolony by OxO treatment (**e**) and source hive (**f**). Line indicates daily mean, shaded area represents ± standard error of the mean

Daily pollen use (Fig. 2a, b) generally decreased over time in all microcolonies. The results of the repeated measures ANOVA showed that daily pollen use was not significantly different by OxO treatment (*p* = 0.540), but was significant by source colony (*p* = 0.002) and time (*p* < 0.001), and the only significant interaction was between source colony*time (*p* < 0.001). There was no significant difference in average daily pollen use between OxO treatments (*p* = 0.152), and no significant interaction between OxO treatment*time (*p* = 0.754), but there was a significant difference between microcolonies started from different source colonies (*p* = 0.004) and a significant interaction between source colony*time (*p* < 0.001), mainly due to the high performance of source colony A. There was no significant interaction between OxO treatment*source colony (*p* = 0.864). Total nectar consumption (as measured by number of sucrose refills per microcolony) was not significantly different between OxO treatments (*p* = 0.536), but was significantly different between microcolonies started from different source colonies (*p* = 0.006).

### Nest building

Nectar and egg cells were counted daily throughout the experiment, and daily average construction rates were calculated. Construction of nectar cells (Fig. 2c, d) was not significantly different between OxO treatments (*p* = 0.991) or by source colony (*p* = 0.959), however, there were significant interactions between OxO treatment*time (*p* < 0.001) and source colony*time (*p* < 0.001). We ran an ANOVA on total nectar cells built by day 35 to check for significant differences between OxO treatment (*p* = 0.370) and source colony (0.086) but found they were not significant at α = 0.05, even with the lower rate of nectar cell building in the high OxO treatment.

Construction of egg cells (Fig. 2e, f) was not significantly different between OxO treatments (*p* = 0.781), yet was significant between microcolonies from different source colonies (*p* = 0.039), with microcolonies from Source A producing the most egg cells. There were significant interactions between OxO treatment*time (*p* < 0.001) and source colony*time (*p* < 0.001). We ran an ANOVA on total egg cells built by day 35 to check for significant differences between OxO treatment (*p* = 0.562) and source colony (*p* = 0.029). Only source colony was a significant factor, again likely due to source A’s high output.

### Survival

**Fig. 3:**
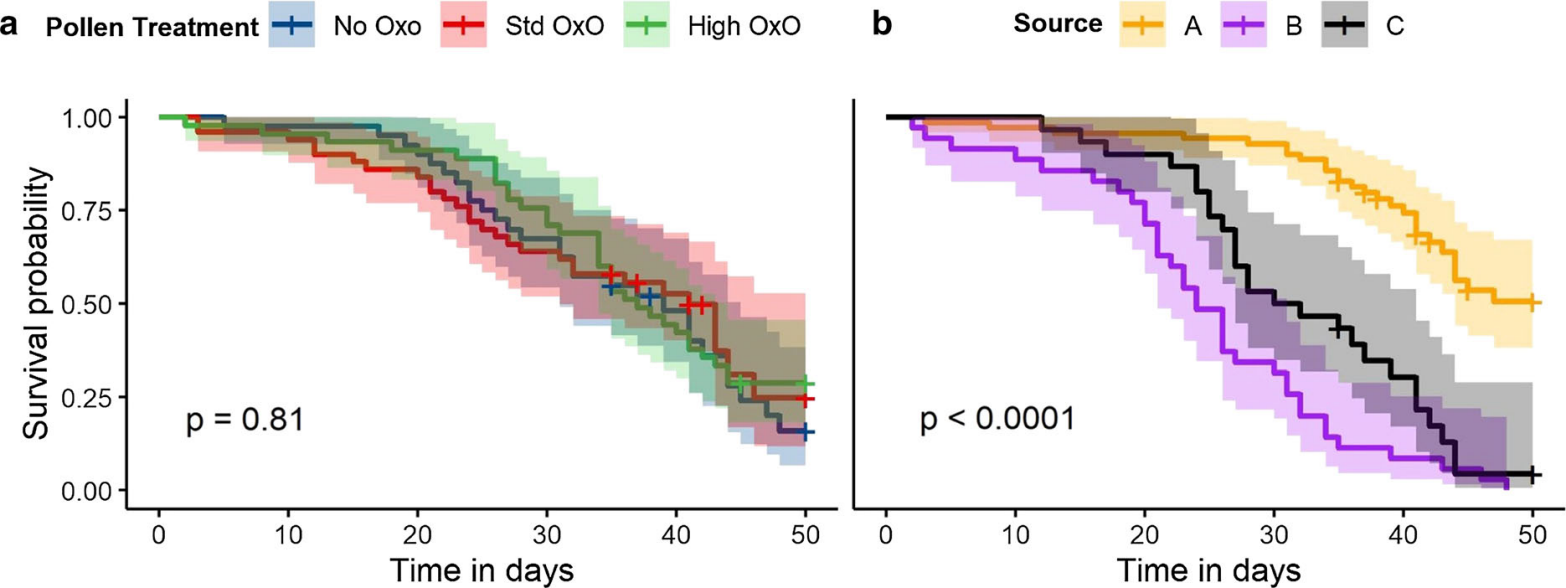
Bee survival by **a** pollen treatment, and **b** source colony. Each step down on the line indicates an individual bee death; ‘ + ’ symbols on charts indicate a microcolony that was censored due to mortality, emergence of adult offspring, or time. Shaded areas indicate 95% confidence interval. Time is number of days after microcolony was filled and treatment started

**Fig. 4:**
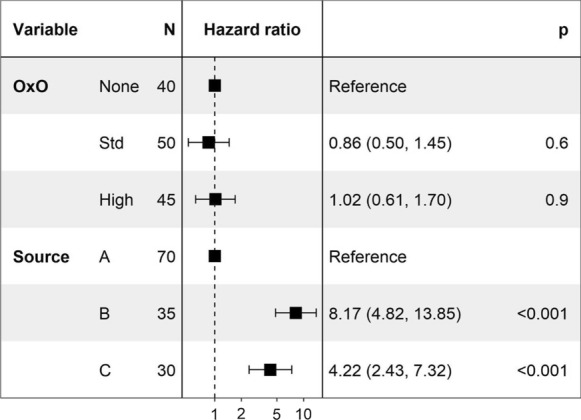
Cox hazard ratio, showing that pollen treatment is not a significant hazard for bee survival. Higher hazard ratios indicate more hazardous conditions in terms of increased mortality. “OxO” indicates OxO concentration in pollen treatment; “Source” (A, B, C) indicates source colony (hive)

Bee mortality over the course of the study was similar for all three pollen treatments (Fig. 3a, no significant differences by treatment; survival differential *p* = 0.81). Mean mortality *was* significantly different between bees from different source colonies, as shown in Fig. 3b (Source A showed lower mortality rates; survival differential *p* < 0.001). The Cox Hazard Ratio (Fig. 4) indicates that neither pollen treatment introduced a significant risk to bee survival (*p* > 0.6) compared to the no-OxO reference, but there were differences between source colonies (*p* < 0.001).

### Reproductive output

Overall reproductive effort was calculated by combining counts of eggs, larvae (ejected during study and dissected at end), and newly emerged adults in all microcolonies. Counts of eggs and larvae were also analyzed separately. The standard OxO treatment showed nearly the same numbers of offspring as the no-OxO control, and none of the differences between pollen treatments were significantly different (Table 1). Masses of eggs and larvae are shown in Supplemental Table 2; these measurements were also not significant between treatments (*p* ≥ 0.154) with source as a random effect. The difference in reproductive output between source colonies (*p* = 0.004) was much larger than differences between OxO treatments (*p* = 0.156), with all larvae and newly emerged adults and the vast majority of eggs originating from source colony A.

### Bee size

**Fig. 5:**
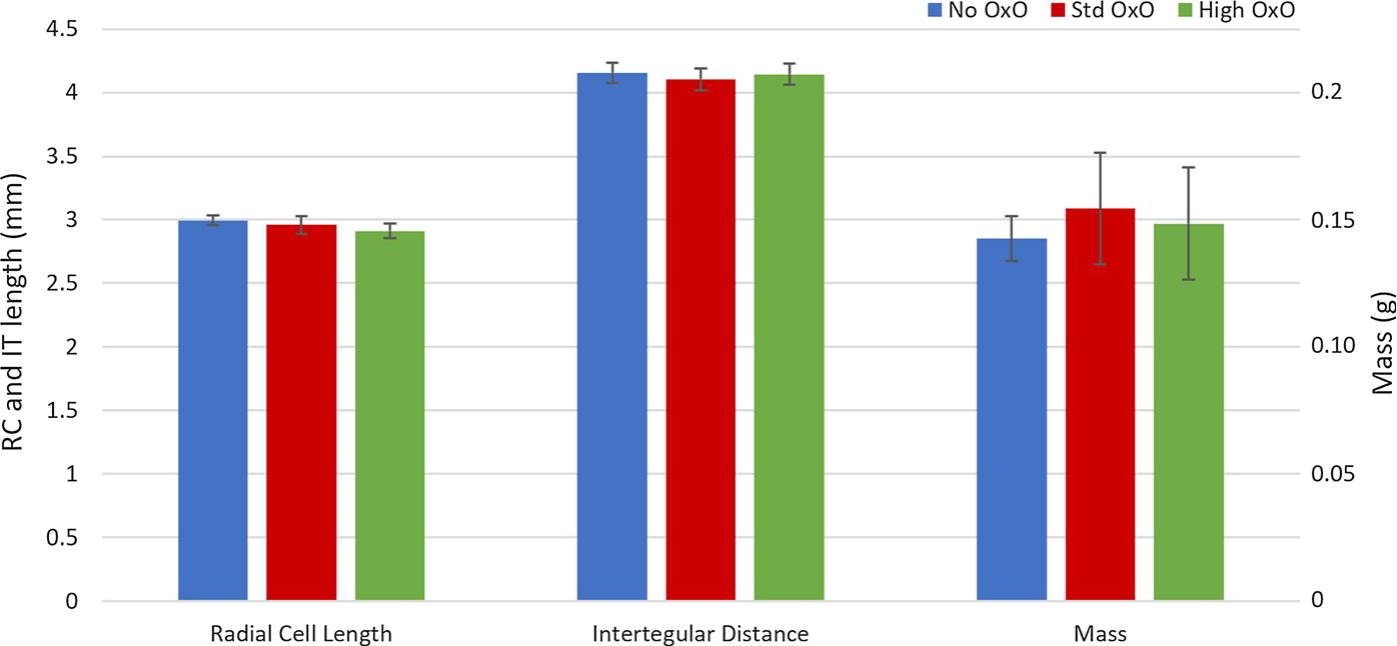
Final Bee Size. “RC” in axis title indicates Radial Cell length; “IT” indicates Intertegular distance. Error bars indicate ± 1 standard error of the mean

Mean final bee size (Fig. 5) did not significantly vary between OxO treatments in terms of mass (*p* = 0.972), intertegular distance (*p* = 0.650), or radial cell length (*p* = 0.647). Mean size was also not significantly different (*p* > 0.087) between bees from different source colonies, but there was a non-significant trend of smaller bees from source colony B compared to sources A and C. Source*treatment interactions were not significant for mass, intertegular distance, or radial cell length analyses in this study.

## Discussion and conclusions

In all analyses of pollen consumption, survival, reproductive output, and size, microcolonies that received pollen with the standard OxO concentration performed similarly to those receiving the no-OxO control. There were non-significant trends of decreased nectar cell construction by bees receiving the artificially high OxO concentration, and slightly increased pollen use by bees receiving the standard OxO concentration. The lack of a dose-dependent correlation and lack of statistical significance suggest these trends may not be biologically important. Additionally, pollen consumption alone is not necessarily an indicator of nutrition or palatability to bees, as pollinators may actually collect more pollen when it is less nutritious, or preferentially select pollen types to meet their nutritional needs (Tasei and Aupinel 2008a; Vaudo et al. 2016). Other analyses (survival, size, early pollen use, and overall egg cell construction) indicated that microcolonies receiving either concentration of OxO in pollen were not different than the no-OxO controls. There were significant treatment*time interactions in the nest building analyses, but cumulative total nectar cells and egg cells at day 35 were not significantly different between treatments. These collective observations suggest that the presence of oxalate oxidase as it will likely be expressed in pollen from transgenic American chestnut trees does not have a detrimental effect on survival, pollen usage, sucrose (nectar) consumption, hive construction, or reproductive effort by bumble bees.

One other observation showed a non-significant trend toward differences between microcolonies that received artificially high concentrations of OxO and no-OxO controls. Counts of total offspring (reproductive effort; Table 1) were negatively correlated with artificially high OxO concentrations, but differences were not statistically significant (*p* > 0.13). Even if these trends are biologically significant, when taken in context of observations on natural secondary metabolite effects on bees (Manson and Thomson 2009; Köhler et al. 2012; Cook et al. 2013; Arnold et al. 2014; Stevenson et al. 2017), this is not an unusual pattern with natural plant defense compounds.

Possibly the most striking differences in this study are between bees from different source colonies. Anecdotal observations from all researchers who worked with the bees in this study confirmed that behavior and productivity were noticeably and consistently different between the source colonies, which is clearly reflected by pollen usage (Fig. 2), survival (Figs. 3 and 4), and reproduction (Table 1). It may be possible to reduce or avoid this source effect in future studies with additional replication, by way of either larger source hives or additional hives. However, there were no strong source*treatment effects in the current study, so the setup of microcolonies arranged by both source hive and treatment accomplished the goal of isolating these effects. Additionally, it is not surprising or unusual that there is variation among bees from different source hives, as genetic diversity would naturally confer variability, and significant source colony differences have been reported previously (Amsalem et al. 2015).

A recurring theme in studies examining toxicological effects on bees is that of “field-realistic” concentrations (Morandin and Winston 2003; Elston et al. 2013; Laycock et al. 2014; Dai et al. 2019). Many compounds, notably including secondary metabolites or alkaloids naturally present in plants, have been observed to negatively affect bee fitness or survival if they are supplied at unrealistically high concentrations, while no detrimental effects (and even some benefits) are seen at realistic concentrations (Manson and Thomson 2009; Köhler et al. 2012; Cook et al. 2013; Arnold et al. 2014; Xavier et al. 2015; Stevenson et al. 2017). For example, nectar rich in the alkaloid gelsemine can deter pollinators or inhibit oocyte development in bees, but at realistic concentrations this compound can reduce the severity of gut parasite infections in *B. impatiens* (Manson et al. 2010). Nicotine (the original compound on which neonicotinoid pesticides are based) and caffeine both naturally occur in some plants and may benefit pollinators at low concentrations, while high concentrations can be inhibitory or even reduce survival of honey bees (Köhler et al. 2012; Stevenson et al. 2017). There are also multiple disease resistance traits naturally found in Asian *Castanea* species (Westbrook et al. 2020b), which could potentially be relevant to native pollinators but were not tested in the current study. These results from natural plant defense compounds provide a valuable context for interpreting the results with OxO.

A few possible mechanisms have been proposed for how OxO expression in plants might affect insect interactions. Most of these do not involve oxalate oxidase directly, but rather the reaction byproduct H_2_O_2_, produced when oxalic acid is degraded by oxalate oxidase. Reactive oxygen species such as H_2_O_2_ can affect insects directly if consumed at sufficient quantities (Ramputh et al. 2002; Zhu-Salzman et al. 2008). Hydrogen peroxide specifically can serve as a signaling molecule within a plant, triggering production of secondary metabolites such as phenolic compounds, which can also affect insect herbivory (Lou and Baldwin 2006; Mao et al. 2007). However, signal cascades and resulting byproducts should not be a factor in this ex situ study, and even *in planta*, pre-germinating pollen is not likely to be a major target of *C. parasitica* infections or plant defense responses. Additionally, hydrogen peroxide is only produced if there is oxalic acid present for the enzyme to act on: chestnut blight is not known to infect flower tissues, and while some plants do store calcium oxalate crystals in flower parts (Barabé et al. 2004), this was not tested in *Castanea* pollen specifically.

Pollinators will likely be exposed to multiple pollen sources in potential restoration scenarios, and bumble bees are generalists that tend to visit multiple pollen or nectar sources consecutively (Babendreier et al. 2008; Goulson 2009), so pollen from any single plant source is not likely in a field-realistic scenario. The nine electron micrographs of mixed pollen from this study showed a mean of 98 identifiable pollen grains per image: a mean of 28% of these pollen grains were identified as chestnut (median 30%, range 4.5–47% across all nine images; median difference between observers was 3.0%). Since the hives used for pollen collection in this study were placed in the midst of several flowering chestnut trees, the ratio of approximately 30% chestnut pollen is likely to be realistic only in areas that are planted intensively with chestnuts, and only during the few weeks in early summer when chestnuts are flowering.

Adequate quantities of pollen were not available directly from transgenic chestnut trees due to limitations imposed by confined field trial permits and the age of available trees, so purified OxO enzyme was applied to non-transgenic chestnut pollen for this experiment. Wheat OxO was not commercially available at the time of this experiment, but barley OxO amino acid sequences share 98% identity with those of the wheat OxO transgene in chestnut (Lane et al. 1993), and both sources show similar enzymatic activity in laboratory assays (Lane 2000; Matthews 2020). Traditional tests to quantify enzyme concentrations in transgenic tissues (Sugiura et al. 1979) were not feasible with currently-available quantities of transgenic pollen, so the standard concentration of OxO in chestnut pollen for this study was chosen to approximately match expression observed in vegetative transgenic chestnut tissues (see below). Preliminary tests of oxalate oxidase activity on limited quantities of pollen from one transgenic event suggest that concentrations in transgenic chestnut pollen are substantially lower than those found in vegetative tissues: quantifying OxO activity on vegetative tissues required dilution of tissue samples to match a standard curve, while no color change was visible in a similar mass of transgenic pollen (Matthews 2020). The concentration of OxO in *vegetative* chestnut tissues of the event known as ‘Darling 58’ is approximately 1 µg OxO/mg fresh weight of transgenic tissue (Matthews 2020; Newhouse et al. 2020). If pollen were collected directly from a transgenic chestnut tree, approximately half would be transgenic (since half of the zygotic cells from a single-copy transgenic parent contain the transgene). This factor and the 30% chestnut ratio in mixed pollen were included in calculations of transgenic chestnut pollen, for a final OxO concentration of 0.15 µg OxO/mg pollen (1 µg OxO/mg transgenic tissue * 30% chestnut pollen * 50% transgenic pollen = 0.15 µg OxO/mg pollen in standard treatment).

The nominally constitutive CaMV-35S promoter that directs expression of the OxO transgene in the current iteration of transgenic chestnuts (Odell et al. 1985; Zhang et al. 2013) has actually been found to express transgenes at very low or negligible levels in pollen from many transgenic plants (Twell et al. 1989; Guerrero et al. 1990; Leede-Plegt et al. 1992; Mascarenhas and Hamilton 1992; Wilkinson et al. 1997; Sunilkumar et al. 2002; Hraška et al. 2008; Jopcik et al. 2014; Yan et al. 2015). This is not surprising, as early-stage pollen cells are essentially dormant until they germinate during pollination, showing relatively lower transcript levels than other tissues, and differentially expressing genes related to germination (Schmid et al. 2005; Pina et al. 2005). Others have observed 35S expression in pollen (Benfey and Chua 1989) and germinating pollen tubes (Fernando et al. 2000), but these studies did not specifically quantify expression relative to other tissues. Only one published source was found that reported observable 35S expression in pollen relative to expression in other tissues from the same plant: some of the tested pollen from some transgenic strawberry events showed expression levels similar to other floral and vegetative tissues (though most pollen from most events showed relatively lower expression) (Mesa et al. 2004). According to these published reports of transgene expression controlled by the CaMV-35S promoter, the highest reported expression level for a protein in pollen is approximately equivalent to expression in leaves, so that was the concentration selected for a conservative standard treatment in the current study.

The above estimates (30% chestnut pollen, 1 µg OxO/mg transgenic tissue) therefore incorporate the highest concentrations of OxO that bees might encounter in potential restoration scenarios. Additionally, the duration of exposure to chestnut pollen in this study (at least 5 weeks) is longer than the duration of ripe chestnut pollen availability under field conditions (approximately 3–4 weeks, personal observations). Therefore, exposure to OxO from transgenic chestnut pollen in field-realistic conditions will likely be lower than even the “standard” concentration tested here. Future studies would be improved if they could be conducted using pollen collected from realistic mixed-species restoration plantings including mature transgenic chestnut trees, but such plantings will not likely be available for at least several years.

Finally, it is worth considering potential benefits of American chestnut restoration to pollinators as well as risks. One study (Tasei and Aupinel 2008a) found that European chestnut (*Castanea sativa*) pollen contained comparatively high levels of nitrogen, considered an indicator of nutritious pollen, and performed the best of 6 species of pure pollen tested for growth and reproduction of *Bombus terrestris*. Since American chestnuts were such prominent members of eastern US forests before blight (Russell 1987), it is not surprising that valuable relationships could have evolved with native insects. Recent observations of several bee species visiting catkins of surviving native *Castanea* species suggest that bees would take advantage of this resource if it were more widespread (Tumminello 2016; Zirkle 2017). Intended American chestnut restoration plans would emphasize planting on disturbed sites or partially cleared areas, similar to existing public and private forest improvement techniques that involve mixed species rather than pure stands of chestnuts (Clark et al. 2014; Westbrook et al. 2020a). Coupled with the lack of detrimental OxO effects, restored American chestnuts (via transgenesis or other means) could be a beneficial resource for native pollinators.

## Supplementary Information

Below is the link to the electronic supplementary material.

Supplementary file1 (PDF 14 KB)

Supplementary file2 (DOCX 34 KB)

## Data Availability

The datasets generated during and/or analyzed during the current study are available from the corresponding author on reasonable request.

## Code availability

Code available upon request.
