# Supplementary material for: Bumble bee (Bombus impatiens) survival, pollen usage, and reproduction are not affected by oxalate oxidase at realistic concentrations in American chestnut (Castanea dentata) pollen
Source: Transgenic Res. 2021 Jun 10;30(6):751–64. doi: 10.1007/s11248-021-00263-w (PMC8580921; doi:10.1007/s11248-021-00263-w)
Supplement: Supplementary file 1 — Supplementary file1 (PDF 14 KB) [file 11248_2021_263_MOESM1_ESM.pdf]

Supplemental Table 1

Start and end dates for each microcolony, with cause for end date. Start date was not a significant factor for pollen use ($p = 0.293$) or combined offspring ($p = 0.217$). Key to end cause: Offsp = adult offspring emerged, Mort = all bees died, 50d = reached 50 days old, Time = Observations stopped on Dec 23.

	**Standard OxO**		**High OxO**		**No OxO Control**	
	Start Date	End Date, Cause	Start Date	End Date, Cause	Start Date	End Date, Cause
Source Colony A	10/24<br>10/28<br>10/31<br>11/3<br>11/11	11/28, Offsp<br>12/5, Offsp<br>12/20, 50d<br>12/16, Offsp<br>12/23, Time	10/27<br>10/29<br>11/2<br>11/7<br>11/11	12/10, Mort<br>12/18, 50d<br>12/22, 50d<br>12/23, Time<br>12/23, Mort	10/28<br>10/31<br>11/2<br>11/9	12/17, 50d<br>12/20, 50d<br>12/11, Offsp<br>12/16, Mort
Source Colony B	10/24<br>10/28<br>11/17	11/16, Mort<br>12/13, Mort<br>12/11, Mort	10/26<br>10/29	11/29, Mort<br>12/2, Mort	10/27<br>10/31	11/29, Mort<br>12/18, Mort
Source Colony C	10/27<br>11/9	12/10, Mort<br>12/23, Mort	10/27<br>10/31	12/17, 50d<br>12/17, Mort	10/29<br>11/17	12/12, Mort<br>12/23, Time
