# Supplementary material for: Bumble bee (Bombus impatiens) survival, pollen usage, and reproduction are not affected by oxalate oxidase at realistic concentrations in American chestnut (Castanea dentata) pollen
Source: Transgenic Res. 2021 Jun 10;30(6):751–64. doi: 10.1007/s11248-021-00263-w (PMC8580921; doi:10.1007/s11248-021-00263-w)
Supplement: Supplementary file 2 — Supplementary file2 (DOCX 34 KB) [file 11248_2021_263_MOESM2_ESM.docx]

**Supplemental Table 2.** Egg and larval mass. Presented as mean ( $\bar{x}$ ) mass per microcolony ±1 standard error of the mean (SEM), with F and p-values from ANOVA. OxO concentration was not a significant factor for the mass of either eggs (p = 0.154) or larvae (p = 0.289). Source colony was included a random effect.

|  | | **Egg mass (g)** | | **Larval mass (g)** | |
| --- | --- | --- | --- | --- | --- |
|  |  | $\bar{x}$ ± SEM | F,  p | $\bar{x}$ ± SEM | F,  p |
| **OxO conc.** | None  (n = 8) | 0.002 ± 0.001 | 2.040, 0.154 | 0.075 ± 0.065 | 1.313, 0.289 |
|  | Std.  (n = 10) | 0.0063 ± 0.0035 |  | 0.085 ± 0.046 |  |
|  | High  (n = 9) | 0.0009 ± 0.0006 |  | 0.0021 ± 0.0019 |  |
